# A DNA Polymerase α Accessory Protein, Mcl1, Is Required for Propagation of Centromere Structures in Fission Yeast

**DOI:** 10.1371/journal.pone.0002221

**Published:** 2008-05-21

**Authors:** Toyoaki Natsume, Yasuhiro Tsutsui, Takashi Sutani, Elaine M. Dunleavy, Alison L. Pidoux, Hiroshi Iwasaki, Katsuhiko Shirahige, Robin C. Allshire, Fumiaki Yamao

**Affiliations:** 1 Division of Mutagenesis, National Institute of Genetics, Mishima, Shizuoka, Japan; 2 Laboratory of Genome Structure & Function, Center for Biological Resources and Informatics, Tokyo Institute of Technology, Yokohama, Kanagawa, Japan; 3 Wellcome Trust Centre for Cell Biology, Institute of Cell Biology, School of Biological Sciences, The University of Edinburgh, Edinburgh, Scotland, United Kingdom; 4 Division of Molecular and Cellular Biology, International Graduate School of Arts and Sciences, Yokohama City University, Yokohama, Kanagawa, Japan; Texas A&M University, United States of America

## Abstract

Specialized chromatin exists at centromeres and must be precisely transmitted during DNA replication. The mechanisms involved in the propagation of these structures remain elusive. Fission yeast centromeres are composed of two chromatin domains: the central CENP-A^Cnp1^ kinetochore domain and flanking heterochromatin domains. Here we show that fission yeast Mcl1, a DNA polymerase α (Polα) accessory protein, is critical for maintenance of centromeric chromatin. In a screen for mutants that alleviate both central domain and outer repeat silencing, we isolated several *cos* mutants, of which *cos1* is allelic to *mcl1*. The *mcl1-101* mutation causes reduced CENP-A^Cnp1^ in the central domain and an aberrant increase in histone acetylation in both domains. These phenotypes are also observed in a mutant of *swi7^+^*, which encodes a catalytic subunit of Polα. Mcl1 forms S-phase-specific nuclear foci, which colocalize with those of PCNA and Polα. These results suggest that Mcl1 and Polα are required for propagation of centromere chromatin structures during DNA replication.

## Introduction

The kinetochore is a multi-protein complex that assembles at the centromere and mediates attachment of chromosomes to spindle microtubules to ensure accurate chromosome segregation at mitosis. The kinetochore is assembled on specialized chromatin containing the histone H3 variant CENP-A and this domain is flanked by pericentric heterochromatin. Pericentric heterochromatin–typified by underacetylation of histone tails and methylation of lysine 9 of histone H3–is bound by heterochromatin protein 1 (HP1) and is required for the recruitment of a high density of cohesin and for the cohesion of sister centromeres.

The DNA sequences of centromeres are not conserved between eukaryotes and, indeed, primary DNA sequence appears not to be an absolute determinant of kinetochore position in most organisms [1]–[3]. Thus, propagation of a particular epigenetic mark could play a pivotal role in propagation of the site of kinetochore assembly. A likely candidate for this mark is CENP-A. To precisely transmit genomic information to daughter cells, these chromatin structures must be maintained during the cell cycle. This is especially the case upon DNA replication when these chromatin structures must be disassembled and reassembled (for review, see [4]). One possibility is that parental CENP-A is redistributed to daughter strands during DNA replication, and the gap is immediately replenished by newly synthesized CENP-A, to reestablish a functional kinetochore. In this case, tight coupling of CENP-A deposition to replication of centromeric DNA would be required to avoid misincorporation of histone H3. However, in both *S. pombe* and mammalian cells there is evidence that CENP-A can be incorporated at centromeres outside S phase [5]–[9]. In *S. pombe*, expression of Cnp1 peaks in G1-phase prior to canonical histone H3 and centromeres are replicated in early S-phase [10], [11]. In humans, expression of CENP-A peaks in G2 phase [5] and it has been demonstrated that newly synthesized CENP-A is incorporated at centromeres in a discrete window in G1 [12]

Fission yeast centromeres resemble those of vertebrates in that the kinetochore domain is found embedded in pericentric heterochromatin. More precisely, fission yeast centromeres are composed of two domains: the central core region (*cnt*), where the kinetochore assembles and the outer repeat region (*otr*), which is packaged as heterochromatin and bound by the HP1 equivalent Swi6 [13], [14]. *S. pombe* CENP-A (Cnp1) and some histone H3 containing nucleosomes that are dimethylated on lysine 4 of histone H3 are associated with the central core domain. Central domain chromatin structure is distinct; partial digestion with micrococcal nuclease produces a smear pattern when hybridized with a central core probe in contrast to the canonical ladder pattern observed for the rest of the genome. This unusual chromatin structure correlates with a functional state of fission yeast centromeres [11], [15]–[17]. Marker genes inserted into fission yeast centromeres are transcriptionally silenced [18], [19] and alleviation of silencing at either the central core or outer repeat heterochromatin has been correlated with loss of centromere function [19]–[22]. In addition, it has been observed that mutants affecting silencing at the central core have no effect on silencing at the outer repeats. Moreover, most mutants affecting silencing at the outer repeats have little or no effect on silencing at the central core. For example, cells lacking Swi6 display alleviation of silencing at the outer repeats of the centromere with no effect on the central core [19], whereas mutant alleles of the kinetochore component *mis6* alleviate silencing at the central core of the centromere but silencing at outer repeat heterochromatin is unperturbed [23]. Alleviation of central core silencing has been used to identify kinetochore proteins and factors that affect Cnp1 incorporation in the central domain [9], [22]; indeed, mutations in *cnp1* itself lead to alleviation of central core silencing [24].

Both central core and heterochromatin domains and their associated proteins are required for full centromere activity [25]. The majority of mutants that alleviate silencing (and concomitantly function) of the centromere are specific for only one of the domains. In an effort to probe the functional relationships between the two domains and the two types of chromatin we implemented a screen to identify *cos* mutants (central core and outer repeat silencing) which alleviate both central core and outer repeat silencing. From this screen, several *mcl1* mutants were identified. We have further analyzed the role of Mcl1 and its binding partner, Swi7 that is a catalytic subunit of DNA polymerase α (Polα), in centromere chromatin structures. The *mcl1* and *swi7* mutants show defective chromatin structure and impaired CENP-A association at the kinetochore domain. Importantly, acetylation of N-terminus tail of histone H4 was aberrantly increased in *mcl1* and *swi7* mutants, and some of *mcl1* phenotypes were partially suppressed by overexpression of some histone deacetylase genes. Finally, Mcl1 and Swi7 formed S-phase foci in the nucleus that overlapped with the replication processivity clamp PCNA. These results suggest that Mcl1 and Polα maintain the hypoacetylated state of kinetochore domain leading to the efficient kinetochore reassembly during DNA replication. We also observed alleviation of transcriptional gene silencing and increased acetylation of histone H4 at the heterochromatin domain, providing an important role of DNA replication machinery in epigenetic inheritance of centromere structures.

## Results

### Isolation of central core and outer repeat silencing (*cos*) mutants

**Figure 1:**
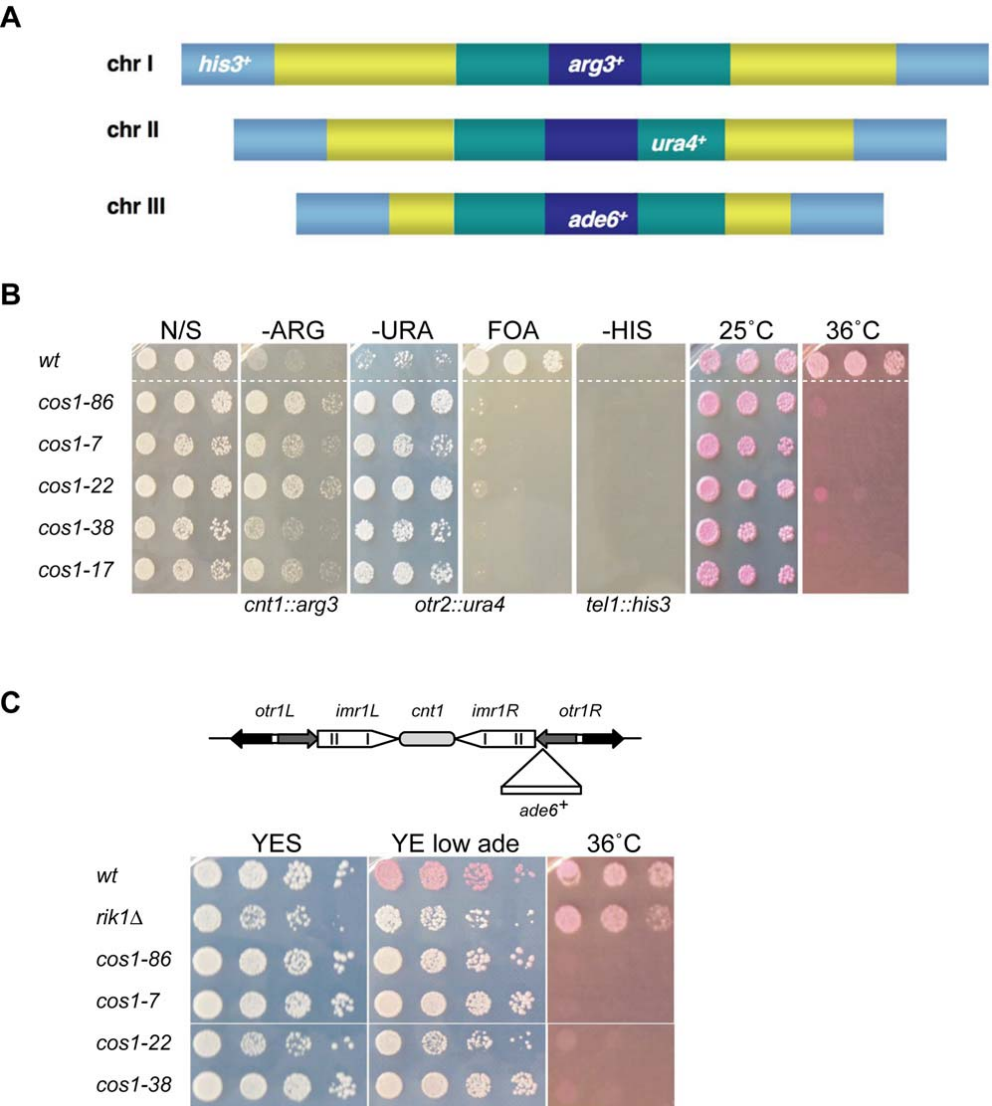
*cos1* mutants alleviate central core and outer repeat silencing. (A) Schematic representation of FY3027 used to isolate mutants defective in central core and outer repeat silencing. FY3027 contains *arg3* ^+^ at the central core of centromere 1 (dark blue) to monitor alleviation of central core silencing [*cnt1:arg3* ^+^], *ura4* ^+^ at the outer repeat of centromere 2 (green) to monitor alleviation of outer repeat silencing [*otr2:ura4* ^+^], *his3* ^+^ at telomere 1 (light blue) to monitor alleviation of silencing at telomeres [*tel1L:his3* ^+^] and *ade6* ^+^ at the central core of cen3 [*cnt3:ade6* ^+^] [22]. Note that the *ade6* ^+^ marker gene was not used for analysis. (B) Tenfold dilutions were plated onto non-selective (N/S) EMM or appropriate plates. *cos1* mutant alleles alleviate silencing at the central core and outer repeats but do not affect silencing at the telomere. *cos1* mutants are temperature sensitive for growth and fail to form colonies on Phloxin B at 36°C. (C) *cos1* mutants alleviate silencing of *ade6* ^+^ gene inserted at *otr1R*. (upper panel) Schematic diagram of the *ade6* ^+^ gene inserted at the outer repeats of centromere 1. (lower panel) Strains were plated on low adenine supplemented YES plates. In wild-type cells (FY1180), *ade6* ^+^ is silenced and colonies are red in colour. *cos1-86*, *cos1-7*, *cos1-22* and *cos1-38* were found to partially alleviate silencing at the outer repeats and were pink in colour.

In order to identify candidate factors required for the propagation of chromatin states at centromeres, a screen was performed to isolate fission yeast mutants that alleviate silencing of both central core (kinetochore) and outer repeat (heterochromatin) domains of the centromere. UV-mutagenesis was performed on a strain containing different marker genes inserted at various silent chromatin domains: the central core of centromere 1 (*cnt1:arg3^+^*), the outer repeat of centromere 2 (*otr2:ura4^+^*), respectively, and a telomere (*tel1L:his3^+^*) (Figure 1A) [22]. Mutants were identified that grew faster than wild-type on both -Arg plates and -Ura plates, but did not grow on -His plates, indicating that central core and outer repeat silencing was alleviated, but telomeric silencing remained intact. Thirteen *cos* (central core and outer repeat silencing) mutants were isolated which fell into 5 complementation groups, *cos1* to *cos5* (data not shown). Further phenotypic characterization of the *cos1* mutants was performed. All *cos1* mutants are temperature sensitive and fail to form colonies at 36^o^C (Figure 1B). *cos1* mutants alleviate silencing of the *ura4^+^* marker in the centromere outer repeat of *cen2* as evidenced by increased growth on -Ura plates; reciprocally, they fail to grow well on the counter-selective drug on 5-fluoroorotic acid (5-FOA). Outer repeat silencing was also assessed in strains in which *ade6^+^* was inserted in centromere 1 (*otr1R*(*Sph*I)*:ade6^+^*); wild-type strains form red colonies on media containing limiting adenine due to transcriptional silencing, whilst heterochromatin mutants such as *rik1*Δ strongly alleviate outer repeat silencing and form white colonies [14], [26]. All *cos1* alleles produced a pale pink colour in this assay suggesting that heterochromatin may not be completely dismantled in these mutants (Figure 1C).

### *cos1* mutants display chromosome segregation defects including lagging chromosomes

**Figure 2:**
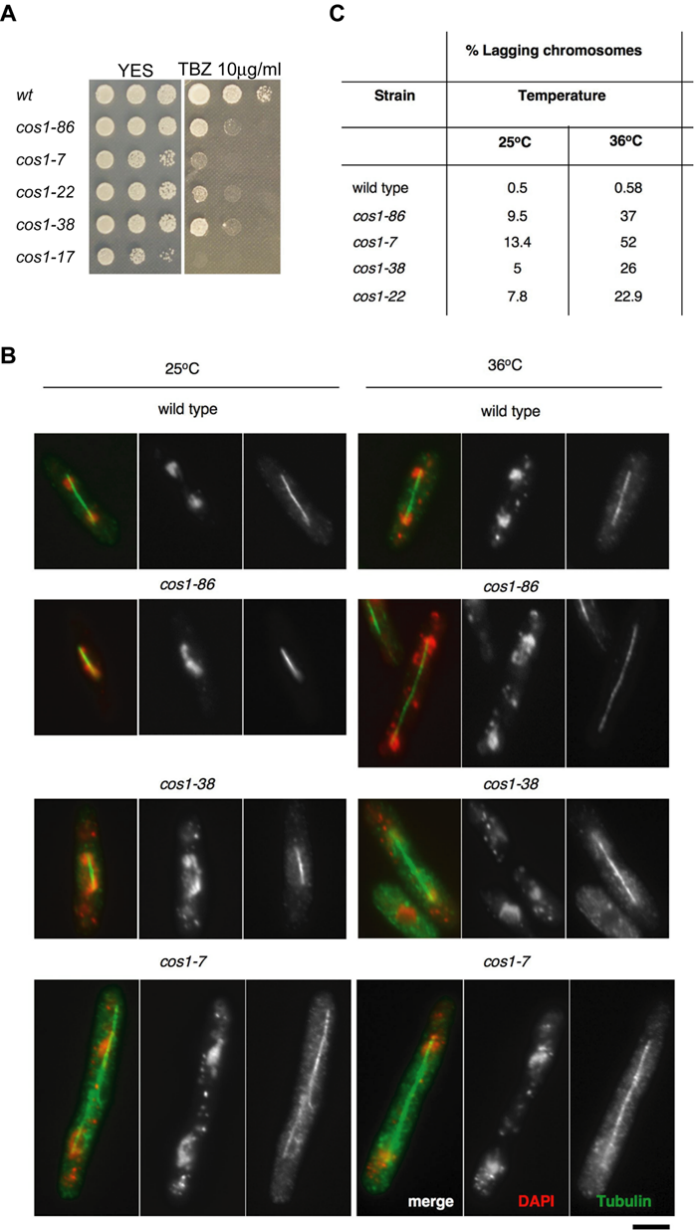
Chromosome segregation defects in *cos1/mcl1* mutants. (A) *cos1* mutants are sensitive to the microtubule destabilizing drug TBZ. Tenfold serial dilutions were spotted onto YES media containing 10 µg/ml TBZ. (B) Wild-type and *cos1/mcl1* mutant cells were grown at 25°C or at 36°C for 6 hours before fixing and staining with anti-α-tubulin antibody (green) and DAPI stained DNA (red). Bar, 3 µm. (C) Frequency of lagging chromosomes in *cos1/mcl1* mutants. Lagging chromosomes were counted in cells with late anaphase spindles and are given as a percentage of total number of cells in anaphase (n = 200). Lagging chromosomes were very rare in wild-type (0.5–0.58%) at both temperatures.

*cos1* mutants were found to show sensitivity to the microtubule-disrupting drug Thiabendazole (TBZ) (Figure 2A). Mutants in centromere function are often impaired in their ability to interact with microtubules and the presence of TBZ exacerbates this defect, reducing cell viability, but has only a very mild effect on wild type growth. Mutant strains that alleviate central core silencing such as *sim4* or outer repeat silencing, such as *swi6*Δ, display defective chromosome segregation, with high rates of lagging chromosomes on late anaphase spindles [19]–[22], [27]. To investigate whether *cos1* mutations result in chromosome segregation defects or abnormal cell morphology, *cos1* mutants and wild-type strain were harvested from log phase cultures grown at permissive and restrictive temperatures (6 hours at 36^o^C). Cells were fixed and stained with anti-α-tubulin antibody to decorate microtubules and 4-,6-diamidino-2-phenylindole (DAPI) to stain for DNA. Cells were then viewed and analyzed for the presence of segregation defects (n = 200). Segregation defects were rarely seen in wild-type strain (approximately 0.5% at 25°C and 36°C). *cos1* mutants displayed multiple forms of chromosome missegregation, including lagging chromosomes at anaphase and unequal segregation of DNA (Figure 2B). The frequency of lagging chromosomes was determined for each *cos1* mutant allele, with *cos1-7* showing the highest degree of segregation defects (13.4% at 25°C and 52% at 36°C) (Figure 2C). Lagging chromosomes and unequal segregation may suggest a role for the *cos1^+^* gene product in establishing correct microtubule-kinetochore attachments or biorientation of the centromere.

### Cloning of *cos1^+^* and identification of *cos1* mutations

*cos1^+^* was cloned by complementation of the temperature sensitivity of *cos1-86* using an *S. pombe* genomic library. The complementing plasmid also reimposed silencing at both centromeric domains, and was found to contain two ORFs: one encoding a putative DNA-directed RNA polymerase III complex subunit, *rpc82^+^* (SPAPB1E7.03) and a 2,548 bp ORF (SPAPB1E7.02) encoding *mcl1^+^* [28]. The entire region covering the *rpc82* and *mcl1* ORFs were sequenced in wild-type and *cos1* mutants. *cos1* mutants were found to have no mutation in the *rpc82* ORF but the following mutations in the *mcl 1* ORF: *cos1-7*, G242R (G to A mutation); *cos1-17*, V233E (T to A mutation); *cos1-22*, A574D (G to A mutation); *cos1-38*, G622E (G to A); *cos1-86*, Q765STOP (C to T), indicating that *cos1* is allelic to *mcl1*.

Mcl1 was previously identified in a screen for mutants that exhibit frequent mini-chromosome loss. Mcl1 is a homologue of budding yeast Ctf4 and is conserved from yeast to human and members of this family contain WD40 repeats in their N-terminus that might provide a protein-protein interaction surface. The *mcl1-1* mutant shows aberrant mitosis such as unequal segregation and lagging chromosomes, and exhibits a defect in sister chromatid cohesion at centromeres that holds sister kinetochores together until anaphase [28]. *cos1* mutants isolated here showed similar phenotypes (Figure 2).

In parallel, we performed a screen for mutants that are synthetically lethal with the null mutation of the *rad2* ^+^ gene (*slr*). Rad2 is the *S. pombe* homologue of Rad27/FEN1 nucleases involved in Okazaki fragment processing during lagging strand synthesis. Among these *slr* mutants, the *slr3-1* was found to be a mutation in the *mcl1* gene (*mcl1-101*) and this mutant showed sensitivity to DNA damaging agents such as MMS and HU. In addition, the *mcl1^+^* gene displayed genetic and physical interactions with the *swi7* ^+^ gene that encodes the largest subunit of Polα, suggesting Mcl1 is involved in lagging strand DNA synthesis [29].

### Silencing is alleviated at the two distinct centromeric domains in *mcl1-101* and *swi7* mutants

**Figure 3:**
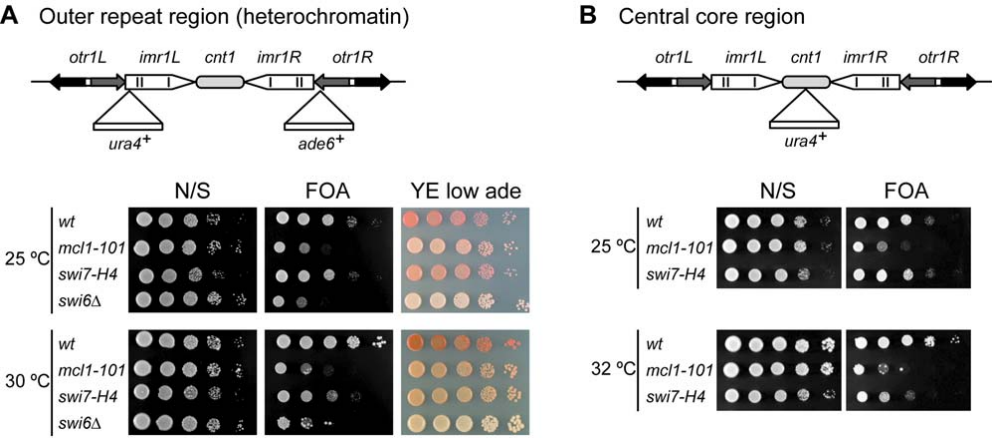
The *mcl1-101* and *swi7-H4* mutants display alleviation of transcriptional gene silencing at both central core and outer repeat regions. (A) (upper panel) The schematic diagram of *ura4* ^+^ and *ade6* ^+^ genes inserted into *imr1L* and *otr1R*, respectively. Note that the *ura4* ^+^ gene is inserted into heterochromatic domain, which is defined as outside of tRNA genes (vertical lines) [23]. (lower panel) Tenfold serial dilutions were spotted. (B) (upper panel) The schematic diagram of the *ura4* ^+^ gene inserted into *cnt1*. (lower panel) Tenfold serial dilutions were spotted.

To determine whether the *mcl1-101* has defects in centromeric silencing like the other *mcl1* mutant alleles, we introduced the *mcl1-101* mutation into indicator strains. Strain FY1193 harbors the *ura4* ^+^ and *ade6* ^+^ genes inserted in the outer repeat *imr* and *otr* elements of *cen1*, respectively [19]. The *mcl1-101* mutant harboring maker genes at the centromere was more sensitive to 5-FOA and was pink on YE low adenine plate compared to the wild-type strain which gave a red color (Figure 3A). The level of alleviation in *mcl1-101* cells was comparable to that of *swi6Δ* cells at *imr* (FOA), but lower at *otr* (low ade). Previously we have revealed strong interaction between *mcl1-101* and *swi7-H4,* a temperature sensitive allele of *swi7^+^* [29]. Therefore we tested transcriptional gene silencing at heterochromatic regions in this mutant. As reported previously [30], *swi7-H4* cells showed alleviation of transcriptional gene silencing at heterochromatic regions (Figure 3A). No dramatic reduction of the heterochromatic marks Swi6 or H3K9me2 on centromeric sequences was apparent in *mcl1* mutants (T.N., E.D., A.P., Y.T., and R.A., unpublished data). Therefore, Mcl1 might function at this domain either independently or downstream of Swi6. Recently, Mamnun *et al*. isolated Mcl1 as an interacting partner of the F-box protein, Pof3, which is a substrate adapter of the SCF ubiquitin ligase and is required for genome integrity. They reported that only a slight change in Swi6-GFP localization could be detected in an *mcl1Δ* mutant [31], consistent with our observations. As the *mcl1-101* mutation genetically interact with *rad2Δ* or *dna2-C2* mutations [29], we examined transcriptional gene silencing in these mutants. However, these mutants did not show any defect in heterochromatin silencing (data not shown), suggesting that Mcl1 and Swi7 might have a specific role in centromeric chromatin structures.

To examine whether the *mcl1-101* and *swi7-H4* mutations affect the maintenance of the chromatin structure at the central core region, each mutation was introduced into *cnt1:ura4* ^+^ strain in which *ura4* ^+^ marker gene is inserted into *cnt1* [19]. As shown in Figure 3B, these mutants were defective in silencing at the kinetochore domain. Thus, both *mcl1-101* and *swi7-H4* mutants exhibit the *cos* phenotype.

### *mcl1* and *swi7* mutants have defects in the specialized chromatin structure at the kinetochore

**Figure 4:**
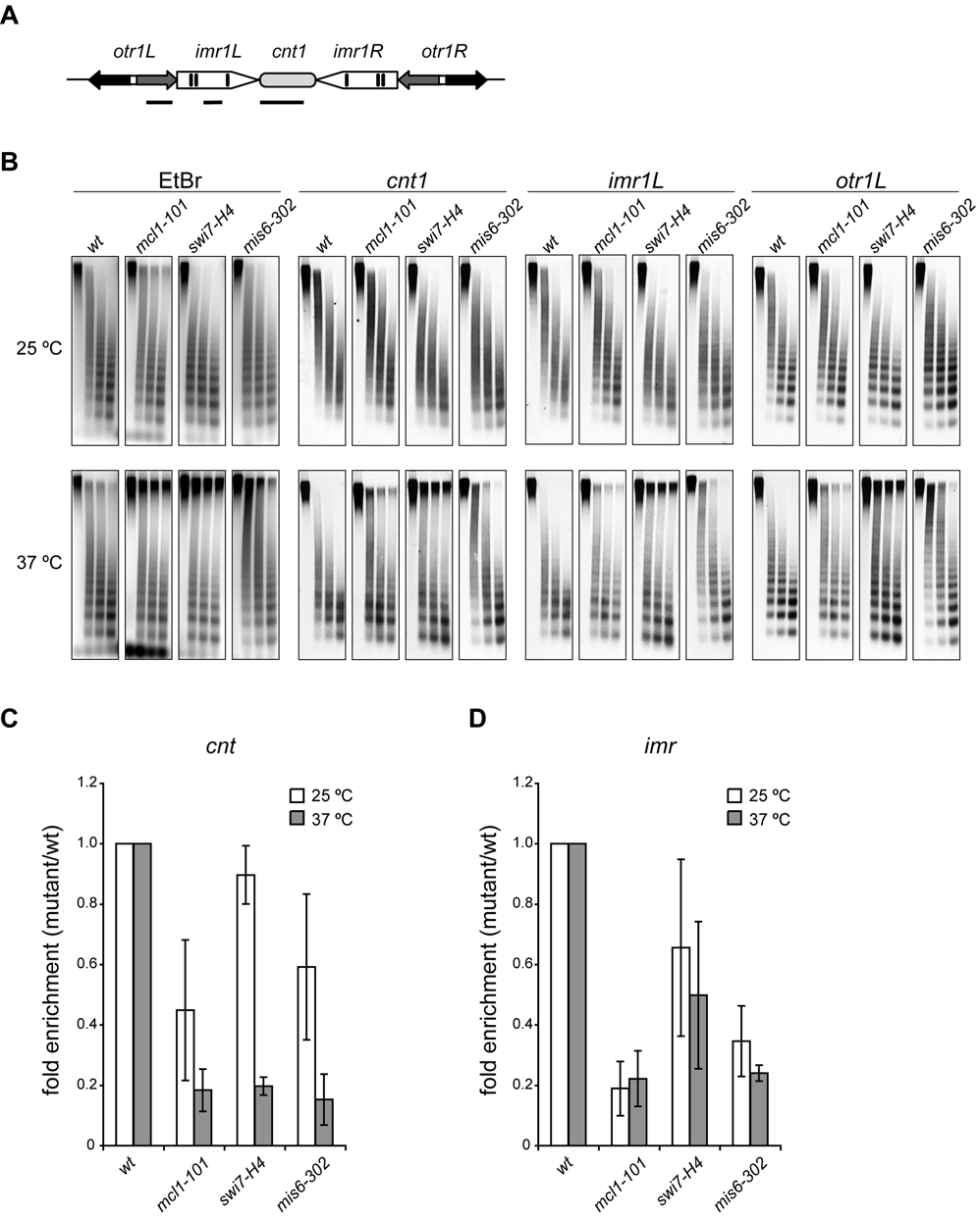
The specialized chromatin structure and association of Cnp1 at central core region is impaired in *mcl1-101* and *swi7-H4* mutants. (A) Locations of probes used in Southern hybridization are depicted as horizontal lines. (B) The cells were harvested from cultures incubated at 25°C or 37°C for 6 hrs and permeabilized by enzyme treatment and chromatin was partially digested with MNase. DNA was extracted, separated on agarose gel, and subjected to Southern hybridization using probes shown in (A). Smeared digestion patterns characteristic of central core region (*cnt1* and *imr1L*) were partially replaced with ladder patterns in *mcl1-101* and *swi7-H4* mutants. Strains were wild-type (JY746), *mcl1-101* (NYSPC41), *swi7-H4* (TN310) and *mis6-302* (NYSPL59). (C) ChIP was performed using the antiserum raised to Cnp1. The cells were incubated at 25°C or 37°C for 6 hrs and then fixed. The ratio of ChIPed DNA to input DNA was calculated as described in Materials and methods and average fold enrichment compared to wild-type strain from three experiments is shown. Localization of Cnp1 was decreased even at permissive temperature in the *mcl1-101* mutant, and was further decreased at restrictive temperature. Decreased Cnp1-association was also seen at 37°C in the *swi7-H4* mutant. Error bars represent the standard deviation. Strains were same as described in (B).

The chromatin structure of the central core domain is known to be unusual since partial micrococcal nuclease (MNase) digestion results in a smeared pattern, rather than the ladder pattern that is indicative of regular nucleosomal packaging [15], [17]. This specialized structure is only associated with a functional centromere context [16]. Importantly, it is reported that integrity of this structure correlates with that of central core silencing [22]. To determine whether Mcl1 and Polα are required for the specialized chromatin structure, chromatin was partially digested with MNase and subjected to Southern hybridization (Figure 4B). In wild-type cells, a typical nucleosomal ladder was observed with a probe corresponding to the heterochromatic domain, outer repeat (*otr1L,* Figure 4A). In contrast, smeared digestion patterns were detected with probes to central core domain (*cnt1* and *imr1L,* Figure 4A), although wild-type cells shifted to 37°C showed a slightly ladder-like pattern. In *mcl1* cells, the smeared patterns of *cnt1* and *imr1L* were partially replaced with a ladder pattern at permissive temperature and the ladder-like pattern of *imr1L* was significantly enhanced at 37°C compared to wild-type cells. Similar results were obtained in *swi7* cells, but only at 37°C. Complete replacement of the smeared pattern was observed in *mis6-302* cells at 37°C as reported previously [32]. These data suggest that Mcl1 and Polα are necessary for assembly of the specialized chromatin structure at the central core domain.

### Genetic interactions with kinetochore mutants

To further investigate the involvement of Mcl1 in the kinetochore function, we examined genetic interactions with kinetochore mutants. *S. pombe* Cnp1 is essential for faithful chromosome segregation and constitutively associates with the centromere central domain to assemble unique nucleosomal structure [11]. The *cnp1-FH* allele was obtained by tagging C-terminus of endogenous *cnp1^+^* gene with 3x FLAG and 6x His (FH). The *cnp1-FH* strain is viable but shows slow growth and sensitivity to TBZ (data not shown). The *mcl1-101* mutant was crossed with the *cnp1-FH*, but the double mutant could not be recovered even at permissive temperature, 25°C (Figure S1A), suggesting that *mcl1-101* and *cnp1-FH* are synthetically lethal.

Next, we examined genetic interactions with mutants deficient in Cnp1 loading to kinetochores. In fission yeast it has been proposed that two distinct pathways mediate Cnp1 loading: a Mis6-dependent pathway and an Ams2-dependent pathway [7]. Mis6 is a homologue of vertebrate CENP-I that constitutively localizes to kinetochores and is required for Cnp1 loading during S and G2 phase [32]. Although the *mcl1-101* mutation was viable in combination with a temperature sensitive allele of *mis6* ^+^, *mis6-302*, the double mutant showed a decreased permissive temperature compared to each single mutant (Figure S1B). Ams2 is a cell cycle regulated GATA factor required for Cnp1 loading during S phase [33]. Synthetic lethality was observed between *mcl1-101* and a null allele of *ams2* (Figure S1C).

Mis16 is a homologue of human RbAp46 and RbAp48, which are found in some histone deacetylase (HDAC) complexes and CAF-1 complex. Mis18 is required for histone hypoacetylation at the kinetochore domain and for the Mis6-dependent Cnp1-loading pathway [34]. *mis16* was tagged with myc at the endogenous locus (*mis16-myc*) and showed no detectable phenotype such as temperature sensitivity or TBZ sensitivity (data not shown). However, construction of a *mis16-myc mcl1-101* strain revealed that myc-tagged Mis16 is not able to cover all the functions of the wild-type Mis16 as the strain showed a drastic decrease in the growth permissive temperature (Figure S1D), indicating that *mis16* and *mcl1* have a synthetic phenotype. Thus, Mcl1 has close functional relationships with Cnp1 and factors involved in its deposition.

### Impaired association of Cnp1 to central core region in *mcl1* and *swi7* mutants

Impaired chromatin structure (Figure 4B) and genetic interactions with Cnp1 and its loading factors (Figure S1) suggested that Mcl1 might be required for Cnp1 association with the central core region. To determine whether Cnp1 association is impaired in *mcl1* cells, chromatin immunoprecipitation (ChIP) was performed using antiserum raised to Cnp1 (Figure 4C). The fraction of *cnt* DNA found in Cnp1 immunoprecipitates was 2-fold lower, even at permissive temperature, in the *mcl1* mutant compared to wild-type, and was further decreased at restrictive temperature. Decrease in *cnt* DNA in Cnp1 immunoprecipitates was also observed in the *swi7* mutant only at restrictive temperature. The fraction of *imr* DNA was severely decreased regardless of growth temperature in the *mcl1* mutant and, to a lesser extent, in the *swi7* mutant.

Loss of Cnp1 from the central core is accompanied by increase of histone H3 in the *ams2Δ* mutant [33], indicating that histone H3 is misincorporated instead of Cnp1. ChIP was also performed using antibody against histone H3. *cnt* and *imr* sequences in histone H3 immunoprecipitates were increased in the *mcl1* and *swi7* mutants indicating that histone H3 is misincorporated into central core region in these mutants (Figure S2). These observations suggest that Mcl1 and Polα are important for efficient Cnp1 incorporation into chromatin of the central core domain. Previous observations that an *mcl1Δ* mutation affected formation of Cnp1-GFP foci at centromeres also support our conclusion [31].

### Aberrant acetylation of histone H4 at centromere in *mcl1* and *swi7* mutants

**Figure 5:**
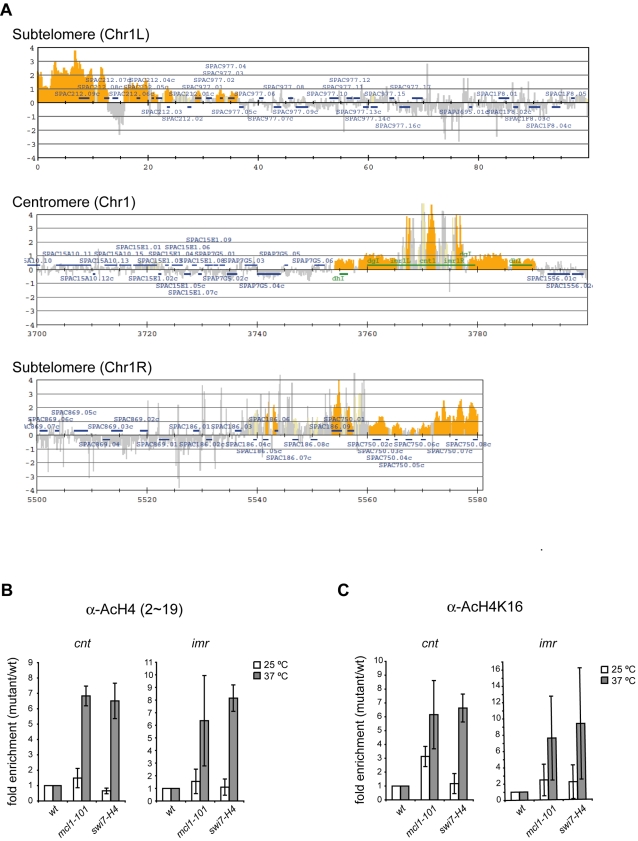
Acetylation of histone H4 is aberrantly increased in the central kinetochore domain in *mcl1* and *swi7* mutants. (A) ChIP-on-chip was performed using the antiserum raised to a peptide corresponding to amino acid 2–19 of histone H4 that is acetylated at K5, K8, K12, and K16 (AcH4-KN). The cells were prepared as described in Figure 4C. Values obtained from AcH4-KN in the *mcl1* mutant were divided by those in wild-type strain. The orange shading represents the binding ratio of loci that show significant enrichment as described previously [68]. Representative results for subtelomeric and centromeric regions of chromosome I are shown. (B,C) ChIP was performed using AcH4-KN (B) and AcH4-K16 (C), respectively. The cells were prepared as described in Figure 4C. Values obtained in the mutants were normalized to those obtained in wild-type strain. Values are further normalized to euchromatic *lys1* locus. Average fold enrichment compared to wild-type are obtained from two repetitions. Strains were wild-type (JY879), *mcl1-101* (NYSPC52), *swi7-H4* (TN403).

The acetylation of N-terminal tails of histone H3 and H4 is maintained at a lower level in the central core region than in coding regions. Here, *mcl1* and, to a lesser extent, *swi7* mutants showed sensitivity to the HDAC inhibitor, Trichostatin A (Figure S3), suggesting that Mcl1 and Polα might be required for maintenance of histone hypoacetylation. To examine this possibility, we performed ChIP-on-chip assays using antiserum raised to a peptide corresponding to amino acid 2-19 of histone H4 acetylated at K5, K8, K12, and K16 (H4-KN). We determined the acetylation status of histone H4 across the entire genome (Figure S4A). Acetylation of histone H4 was found at the promoter regions of most genes, whereas H4 was hypoacetylated at centromeric regions. Although acetylation peaks similar to those in wild-type were observed at most genes in the *mcl1* mutant, histone H4 acetylation was elevated at the centromere region in *mcl1* mutant (Figure S4B). Comparison of H4-KN data obtained from *mcl1* and wild-type indicates that acetylation level was increased at centromere and sub-telomeric regions in the *mcl1* mutant (Figure 5A).

To confirm these results, we performed independent ChIP analyses and quantified the precipitated DNA by quantitative real-time PCR (qPCR). Consistent with the ChIP-on-chip data, the level of H4-KN acetylation at the kinetochore domain was increased in the *mcl1* mutant compared to the wild-type strain. A similar increase in acetylation was seen in the *swi7* mutant (Figure 5B). When we used the specific antibody against acetylated H4-K16, acetylation at this residue was also increased in both mutants at restrictive temperature and, to a lesser extent, at permissive temperature in the *mcl1* mutant (Figure 5C). These results suggest that Mcl1 and Polα are required for the hypoacetylated state of the histone H4 N-terminal tail including K16 in the kinetochore domain of fission yeast centromeres.

### Multicopy suppression of *mcl1* phenotypes by histone deacetylase genes

**Figure 6:**
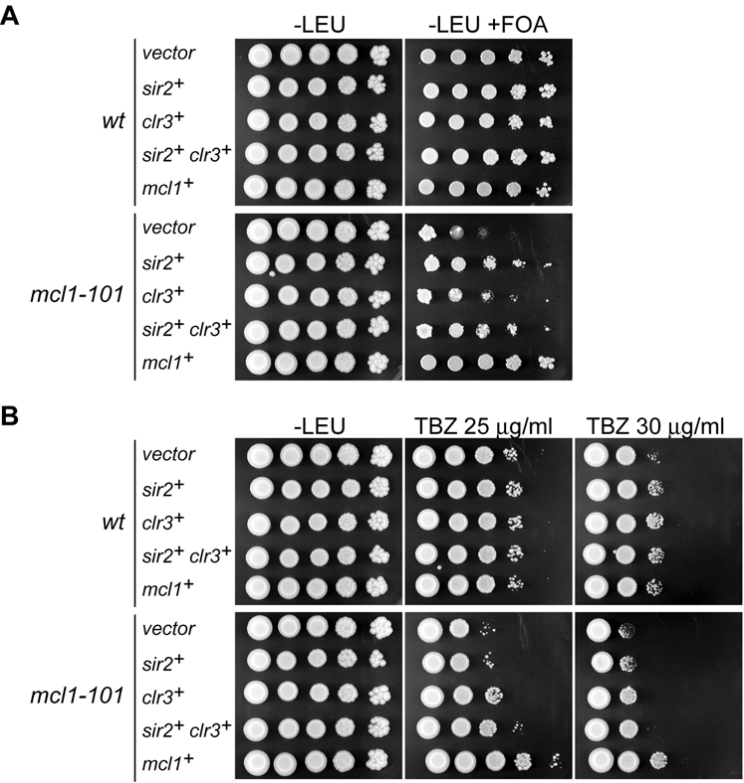
Multi-copy suppression of *mcl1-101* phenotypes by *sir2* ^+^ and *clr3* ^+^ HDAC genes. Wild-type and *mcl1-101* mutant harboring the *ura4* ^+^ insertion at the *cnt1* were transformed with multi-copy plasmid carrying *sir2* ^+^, *clr3* ^+^, or both, respectively. Tenfold serial dilutions were spotted onto EMM-Leu and EMM-Leu containing 5-FOA (A) or EMM-Leu containing indicated concentration of TBZ (B). In panel A, *sir2* ^+^ and, to a lesser extent, *clr3* ^+^ partially suppressed 5-FOA sensitivity of the *mcl1-101* mutant. In panel B, *clr3* ^+^ partially suppressed TBZ sensitivity of the *mcl1-101* mutant, and *sir2* ^+^ also relieved the sensitivity only in the presence of *clr3* ^+^.

Increased acetylation of histone H4 in the *mcl1* mutant raised the possibility that Mcl1 might regulate HDAC(s) to maintain the hypoacetylated state of the kinetochore domain. The *S. pombe* genome encodes six HDACs that belong to three distinct classes. Among these HDACs, Clr3, Clr6 and Hst4 are required for transcriptional gene silencing at the central core region [35], [36]. In addition, the HDAC Sir2 preferentially localizes to the central core region [37]. To examine whether these HDACs are functionally related to Mcl1, multi-copy plasmids carrying each of the HDAC genes were introduced into *cnt1:ura4^+^ mcl1-101* cells, and sensitivity to 5-FOA and TBZ, were examined. As shown in Figure 6A, alleviation of silencing in the *mcl1* mutant was partially suppressed by multi-copy plasmids of *sir2^+^* gene and, to a lesser extent, of the *clr3^+^* gene. Furthermore, TBZ sensitivity was partially suppressed by the *clr3^+^* plasmid and simultaneous introduction of *sir2^+^* and *clr3^+^* genes further relieved the sensitivity (Figure 6B). These results suggest that Mcl1 functionally interacts with Sir2 and Clr3 HDACs in maintaining centromere integrity.

### Localization of Mcl1 to replication foci

**Figure 7:**
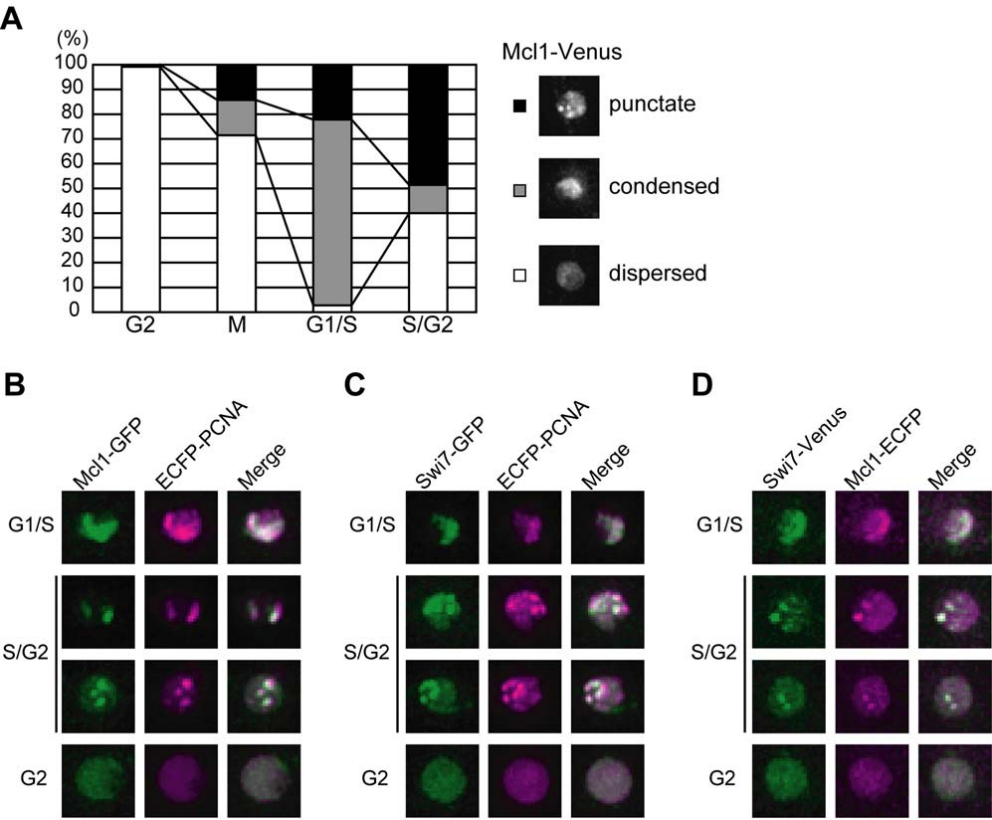
Mcl1 localizes to replication foci during S-phase. (A) Mcl1 changes its nuclear localization during S-phase. Cells expressing Mcl1-Venus from the endogenous locus were classified into 4 cell cycle stages according to their morphology. The percentage of cells showing punctate (closed bar), condensed (shaded bar), and dispersed (open bar) localization of Mcl1-Venus in various cell cycle stages are shown (left panel). Representative photographs of each localization pattern are shown (right panel). (B–D) Mcl1 and Swi7 colocalize with PCNA in the S-phase nucleus. Mcl1-GFP and ECFP-PCNA (B), Swi7-GFP and ECFP-PCNA (C), and Swi7-Venus and Mcl1-ECFP (D) are shown. Merged images are also shown.

Mcl1 was previously shown to be a constitutive nuclear protein that associates with chromatin from G1 to S phase [28]. To further examine the nuclear localization of Mcl1, the endogenous *mcl1^+^* gene was C-terminally tagged with the fluorescent marker Venus [38]. The *mcl1^+^-venus* cell did not show any phenotypes such as HU and MMS sensitivity (data not shown). Mcl1-Venus localized to the nucleus throughout the cell cycle, and a portion of cells showed a punctate (Figure 7A, top) or condensed pattern (Figure 7A, middle) of Mcl1-Venus signal. To determine which stage of the cell cycle shows such localization pattern, *mcl1^+^-venus* cells were stained with Hoechst 33432 to visualize nucleus and septum, and roughly classified into four cell cycle stages (Figure 7A). A diffuse Mcl1-Venus signal was observed in the nucleus of most cells with a single nucleus (G2-phase). Condensed localization of Mcl1-Venus peaked in cells with a septum (G1/S-phase) and decreased in separating cells (S/G2-phase). The punctate localization of Mcl1-Venus increased from G1/S-phase and accounted for about half of S/G2 cells. These results indicate that Mcl1 changes its nuclear localization pattern during DNA replication.

In vertebrate cells, DNA polymerases and their accessory proteins such as PCNA cluster together and form nuclear foci called “replication factories” [39]. Similar nuclear structures are also observed in budding and fission yeasts. In S-phase budding yeast cells, POLα, POLε and PCNA tagged with GFP or EYFP forms globular nuclear signals, and more importantly, DNA replication actually occurs in these foci [40]. Fission yeast PCNA-ECFP also forms foci during DNA replication [41]. Since Mcl1 physically interacts with Polα and associates with chromatin in a G1/S-phase specific manner, we tested whether the Mcl1 foci formed during S-phase corresponds to replication factories. PCNA-ECFP was expressed from its native promoter together with Mcl1-GFP. Multiple nuclear foci of ECFP-PCNA were observed in S-phase cells as previously reported, and these PCNA foci overlapped with those of Mcl1-GFP (Figure 7B). We also examined cells expressing Swi7-GFP and ECFP-PCNA (Figure 7C). Consistent with the physical interaction between them, Mcl1 and Swi7 also colocalized in S-phase nuclei (Figure 7D). Together, these observations strongly suggest that Mcl1 localizes to the replication fork, interacting with Polα. We also examined the relationship between kinetochores and Mcl1 foci formed during S-phase. ECFP-Cnp1 was expressed from *nmt41* promoter in *mcl1* ^+^-*GFP* cells, but the signals did not colocalize in most cells (data not shown), which might reflect the short time window in which centromeres are replicated.

## Discussion

In this study, we identified several *cos* mutants, which alleviate silencing at both the central kinetochore and outer heterochromatin regions of fission yeast centromeres. *cos1* is allelic to *mcl1* which was previously show to be important for chromosome replication, segregation and sister chromatid cohesion. In addition, we demonstrate that a mutant in the catalytic subunit of Polα, Swi7, also shows the *cos* phenotype. In both *mcl1* and *swi7* mutants the levels of the centromeric histone Cnp1 associated with the central domain are reduced and there is partial loss of the unique chromatin structure of this region. Although outer repeat silencing was alleviated, no reduction of the heterochromatic marks Swi6 or H3K9me2 on centromeric sequences was apparent (T.N., E.D., A.P., Y.T., and R.A., unpublished data). Interestingly, the acetylation of histone H4 N-terminal tails was aberrantly increased at both domains. Finally, we showed that Mcl1 co-localized with both PCNA and Swi7 mainly during S-phase. From these results, we propose that Mcl1 and Swi7 are involved in propagation of centromereic chromatin structures during DNA replication by regulating the level of histone acetylation. Our observation that the overexpression of Sir2 and/or Clr3 partially suppressed some of *mcl1* phenotype suggests that Mcl1 might control these HDACs during DNA replication.

### The role of Mcl1 in regulation of specific chromatin structures

Here we show that Mcl1 and Polαhave roles in regulation of specific chromatin structures: the central core and outer heterochromatin regions of fission yeast centromeres. Evidence presented here suggests that Mcl1 may act on central core chromatin by ensuring that Cnp1 chromatin is established and maintained at centromeres during replication. In fission yeast, Cnp1 incorporation into centromeres depends on two distinct pathways in S and G2; Mis6, a homologue of CENP-I in vertebrates, is required in both S- and G2-phase loading [7], [11] and a cell cycle-regulated GATA factor, Ams2, is also critical for Cnp1 loading during S-phase [7], [33]. In addition, Mis16 that is homologous to human RbAp46 and RbAp48, which are found in some histone deacetylase (HDAC) complexes and with histone chaperone CAF-1 [42]–[46], is also required for Cnp1 loading [34]. As the *mcl1-101* mutation is synthetically lethal with both *mis6-302* and *ams2Δ* and also shows genetic interactions with *mis16*, it may be that Mcl1 performs a function in Cnp1 loading that is independent of both S and G2 loading pathways.

Mcl1 may also regulate chromatin at centromeres by influencing the levels of histone acetylation at central core and heterochromatin domains. In fission yeast, a global function in chromatin regulation has also been reported for the CHD (chromo-ATPase/helicase-DNA binding) domain-containing chromatin remodeling factor, Hrp1 [47]. Interestingly, human CHD proteins, CHD3 and CHD4, were co-purified with HDAC1/2 to form a protein complex called NuRD (nucleosomal remodeling histone deactylase), and ATP-dependent remodeling activity is required for deacetylation of oligonucleosomal histones [43]–[45]. The *S. pombe hrp1Δ* mutant, like *mcl1-101*, shows alleviation of silencing at both domains of the centromere and the *mat* locus, and is defective in Cnp1 incorporation into the central core region, concomitant with aberrant acetylation of histone H3 and H4. Notably, Hrp1 localizes to the kinetochore domain in early S-phase when centromeres are replicated [10], suggesting that Hrp1 acts during DNA replication [47]. One possibility is that Hrp1 may enhance histone deacetylation by modulating chromatin structure at the central core region. It is also possible that Mcl1 might control chromatin remodeling in concert with Hrp1, or the HDACs Sir2 and Clr3, to enhance subsequent histone deacetylation.

### Histone deacetylation during DNA replication

Does the removal of histone acetylation during or soon after replication have any importance for the epigenetic inheritance of chromatin structures? This is the case in human cells. In mammalian cells, DNA synthesis occurs at discrete sites in the nucleus called replication foci or replication factories [39]. In early S-phase, numerous foci of pulse labeled BrdU are distributed throughout the nucleus except for nucleoli, and these foci correspond to transcriptionally active chromatin. In late S-phase, replication foci can be found along the nuclear periphery and perinucleolar regions, and finally at a few larger sites, which represent the replication of heterochromatic regions. Interestingly, acetylation at K5 and K12 in histone H4, which is a hallmark of newly synthesized histones, is removed within 20 min after DNA synthesis at late replicating foci, but not early replicating foci, and this immediate deacetylation is inhibited by Trichostatin A treatment [48]. These findings suggest that certain HDAC(s) act(s) only on late replicating foci to couple the incorporation of highly-acetylated histones to subsequent deacetylation events.

Furthermore, in human cells it has been demonstrated that late replication foci contain a HDAC and also that PCNA interacts with the DNA methyltransferase DNMT1, which localizes to both early and late replication foci [49]. Intriguingly, DNMT1 also interacts with HDAC2, and they colocalize only in late replicating foci, suggesting that HDAC activity is preferentially recruited to late replication foci by the replication machinery. We have shown here that Mcl1 colocalizes to S-phase nuclear foci of PCNA, which might correspond to mammalian replication foci. Thus, Mcl1 might regulate the recruitment of a HDAC to the kinetochore domain in certain replication foci, though fission yeast centromeres are known to be replicated early in S-phase. Since the level of histone acetylation is determined by a balance between HDAC and HAT activities, it remains possible that Mcl1 is essential to prevent HAT activity from acting on kinetochore domain.

### Mcl1 and the DNA replication fork

We demonstrate that the Mcl1 nuclear foci formed during S-phase overlap with those of PCNA and Swi7. Although we currently have no direct evidence, we suppose that Mcl1 localizes to the progressing DNA replication fork for the following reasons. Firstly, Mcl1 interacts physically with Swi7 *in vivo* and *in vitro* and binds to chromatin tightly during S-phase [28], [29], [50]. Secondly, the budding yeast homologue, Ctf4, moves along chromosomes with the replication fork as revealed by ChIP-on-chip experiments [51]. Finally Ctf4 is a component of replication progressive complex (RPC) including other replication proteins such as the MCM proteins and the GINS complex [52]. Because we were not able to detect stable colocalization of Mcl1-GFP foci with ECFP-Cnp1 foci in asynchronous culture (data not shown), Mcl1 might localize to centromere chromatin only in a discrete window when centromeric DNA is replicated.

We have previously shown that the *mcl1* mutant shows genetic interactions with mutations defective in Okazaki fragment maturation such as *rad2* (*RAD27*/*FEN1*) and *dna2*, and it accumulates double strand breaks after completion of bulk DNA synthesis, suggesting that Mcl1 is involved in lagging strand DNA synthesis [29]. It is possible that defective centromere chromatin structures observed in *mcl1* and *swi7* mutants might be a secondary effect of an unstable DNA replication fork. However, the *rad2Δ* mutant did not show any defect in kinetochore and heterochromatin silencing (data not shown), suggesting that Mcl1 and Swi7 might have a specific role in maintaining centromeric chromatin structures.

An understanding of the roles of Mcl1 and Swi7 in the maintenance of centromeric chromatin structures and how this relates to their roles in DNA replication remains to be uncovered. The *swi7-H4* allele used in this study (G889D) is located within homology box VI, which is in the conserved nucleotide binding domain found in all DNA polymerases [30]. Another *swi7* mutation, *swi7-1*, maps to the non-conserved C-terminal region and shows a defect in heterochromatic silencing [53]. This mutation also showed alleviation of kinetochore domain silencing (data not shown), suggesting that Swi7 has a role in centromeric chromatin assembly independent of its catalytic activity. All six alleles of *mcl1* show defects in centromeric silencing and we have shown that *mcl1-101* has defects in both DNA replication and maintenance of chromatin structures (this study and [29], [31]). To understand the role of Mcl1 in kinetochore assembly, further experiments such as isolation of separation of function mutations, which show a defect in only maintenance of chromatin structures or DNA replication, might be required. Alternatively, all phenotypes observed in the *mcl1-101* mutant could be attributable to loss of the sole function of Mcl1 (for example, regulation of histone deacetylation) and thus be inseparable.

### Mcl1 and budding yeast Ctf4 have conserved functions in chromosome metabolism

The budding yeast homologue of Mcl1, Ctf4, is required for chromosome stability [54], [55] It physically interacts with Pol1, a catalytic subunit of Polα in budding yeast [56], [57]. Ctf4 travels along chromosomes with the replication fork [51]; deletion of *CTF4* results in premature separation of sister chromatids [58]. These findings suggest that Ctf4 is required for establishment of sister chromatid cohesion during S-phase. Interestingly, deletion of *CTF4* also shows defects in transcriptional silencing at the cryptic mating type locus, *HMR*, and at telomeric regions [59], suggesting that Ctf4 is involved in the regulation of chromatin structures as Mcl1 is. The molecular function of Ctf4, however, remains unclear, and it is intriguing to address whether Ctf4 is implicated in modulating the level of histone acetylation. In addition, Ctf4 competes for binding to Pol1 with the Spt16 subunit of FACT complex, which removes H2A-H2B dimers during transcript elongation and is also required for DNA replication [60]. Deletion of the *S. pombe* FACT subunit Pob3 causes alleviation of silencing at *otr*, although the effects at *cnt* are less clear-cut [61]. Therefore, our observations reported here strengthen the importance of Polα and its accessory proteins, such as FACT and Mcl1/Ctf4, in faithful propagation of chromatin structures during DNA replication.

## Materials and Methods

### Strains, plasmids, and growth conditions

*S. pombe* strains used in this study is listed in Table S1. *S. pombe* cells were routinely grown in YES, EMM or PMG supplemented with appropriate nutrients. Standard genetic manipulations were used as previously described [62]. A *LEU2* ^+^ *S. pombe* genomic library was kindly provided by Chikashi Shimoda. The *mis16^+^*, *cnp1^+^*, *mcl1^+^*, and *swi7^+^* were tagged at their endogenous loci by PCR-based tagging modules as described previously [63]. For C-terminal tagging with the Venus gene, The *Pac*I-*Asc*I fragment containing the *gfp* gene of pFA6a-GFP-kanMX6 was replaced with the Venus gene (a gift from Atsushi Miyawaki) to give pFA6a-Venus-kanMX6. The pFA6a-Flag His (FH)-kanMX6 was kindly provided by Takashi Morishita. The nucleotide sequences of primers used here are listed in Table S2.

For deletion of the *ams2^+^* gene, the genomic *Sac*I-*Hin*dIII fragment containing the *ams2^+^* gene was amplified from 972h^-^ with Ams2-F0 and Ams2-R0, and cloned into *Sac*I and *Hin*dIII sites of pUC19 to give pUC19-Ams2. The 1.4-kb fragment containing was amplified from pFA6a-kanMX6 [63] with KanMX6-F0 and KanMX6-R0, and digested with *Sma*I. The *Bst*XI fragment containing most ORF of *ams2^+^* gene in pUC19-ams2 was replaced with the 1.4-kb P_TEF_-kan^r^-T_TEF_ fragment by blunt-end ligation to give pUC19-ams2::kan^r^. To introduce multi-copy of HDAC genes, the genomic fragments containing *sir2^+^* and *clr3^+^* genes were amplified from 972 *h* ^−^ strain with Sir2-F0 and Sir2-R0, Clr3-F0 and Clr3-R0, respectively. These fragment were digested with *Pst*I (*sir2^+^*) and *Bam*HI (*clr3^+^*), and cloned into *Pst*I and *Bam*HI sites of pSP102 (*ars2004*, *LEU2*) [64] to give pSP102-Sir2 and pSP102-Clr3, respectively. The 3.5-kb *Bam*HI fragment containing the *clr3^+^* gene was excised from pSP102-Clr3, blunt-ended with T4 DNA polymerase, and cloned into the *Sma*I site of pSP102-Sir2 to give pSP102-Sir2-Clr3. The nucleotide sequences of primers used here are listed in Table S2.

### Isolation and cloning of *cos* mutants

Wild-type strain (FY3027 *h^+^*) was plated either onto medium lacking arginine (-Arg) and then onto medium lacking uracil (-Ura) or directly on to double selection medium lacking both arginine and uracil (-Arg, -Ura) and mutagenised by UV (3–5 mJ, 50–80% killing). Plates were incubated at 25^o^C for 5–20 days and fast growing colonies (184 colonies from –Arg plates, 54 colonies from -Arg, -Ura plates) were picked and streaked onto –Arg plates to retest for alleviation of silencing. Fast growing colonies were subsequently picked from these –Arg plates and streaked onto –Ura plates to assay alleviation of silencing at outer repeats (*otr)*. In total, 13 *cos* mutants that alleviate silencing at both the central core and the outer repeat domains were isolated.

In order to clone the *cos1^+^* gene, a *LEU2* ^+^ *S. pombe* genomic library (gift from Chikashi Shimoda) was transformed into *cos1* mutants and cells were screened for rescue of their ts phenotype. Transformants were plated on minimal medium lacking leucine and containing phloxin (0.02% v/v). After 5 days growth at 25°C, plates were shifted to 36°C for 1–2 days, to select for colonies that were now capable of growing at this temperature. At 36°C, 1 single colony out of 18,000 *cos1-86* colonies analysed was pale pink in color and could grow at the restrictive temperature.

### Micrococcal nuclease assay

Micrococcal nuclease assay was performed as described previously [65]. MNase-digested samples were separated on 1.5% agarose gel and analyzed by Southern hybridization using AlkPhos Direct Labelling and Detection System (Amersham). Since the efficiency of MNase digestion varied among strains, samples that show a similar digestion efficiency judged by EtBr staining were used for analysis. The nucleotide sequences of primers used for amplifying probe DNA are listed in Table S2.

### Chromatin immunoprecipitation (ChIP)

ChIP was performed as described previously [66]. Antibodies used were antiserum raised to N-terminal peptide of Cnp1, α-acetyl-histone H4 antiserum (Upstate), α-acetyl-histone H4 (Lys16) antiserum (Upstate), and α-histone H4 pan antibody (Upstate), respectively. Quantification of ChIPed DNA was performed by real-time PCR. The nucleotide sequences of primers used in real-time PCR are listed in Table S2.

ChIP-on-chip was carried out using IVT amplification method as described previously [67]. All the array data are available at GEO database (http://www.ncbi.nlm.nih.gov/projects/geo/) through the accession number of GSE11102.

### Fluorescence microscopy

Cells were grown to mid-log phase and washed with DW and adhered to glass bottom dish coated with lectin. Z-stack images were captured at intervals of 0.3 µm using DeltaVison (Applied Precision). After 3D deconvolution, projected images were used for judging colocalization of two differently tagged proteins. To stain nucleus and septum, cells were treated with 1 µg/ml of Hoechst 33432 for 30 min.

## Supporting Information

Figure S1 The *mcl1-101* mutant interacts with kinetochore mutants genetically. (A) The *mcl1-101* mutation is synthetically lethal with *cnp1-FH*. The *mcl1-101* mutant (NYSPC40) was crossed with *cnp1-FH* (TN705). Resultant asci were dissected and incubated at 25 °C. Open squares indicate locations of double mutant spores. (B) Permissive growth temperature of *mcl1-101* mutant is decreased by the *mis6-302* mutation. Cells were grown at 25 °C and ten-fold serial dilutions were plated onto YES plates. Plates were incubated at indicated temperature for 3 days. Strains were derived from a cross between *mcl1-101* (NYSPC41) and *mis6-302* (NYSPL58). (C) The *mcl1-101* mutation is synthetically lethal with *ams2Δ* mutation. The *mcl1-101* mutant (NYSPC40) was crossed with *ams2Δ* (NYSPK66) as described in A. (D) Permissive growth temperature of *mcl1-101* mutant is decreased by the *mis16-13myc* allele. Strains were wild-type (TN212), *mis16-13myc* (TN968), *mcl1-101* (NYSPC41), *mis16-13myc mcl1-101* (TN1035).
(0.70 MB TIF)

Figure S2 Histone H3 is aberrantly incorporated into kinetochore domain. ChIP was performed using antibody against C-terminal part of human histone H3. The ratio of immunoprecipitated DNA to input DNA was calculated and normalized to that of euchromatic *lys1* locus. Fold enrichment compared to wild-type is shown. Open bars and shaded bars indicate results of 25 °C and 37 °C, respectively. Strains were wild-type (JY879), *mcl1-101* (NYSPC52), *swi7-H4* (TN403).
(0.10 MB TIF)

Figure S3 The *mcl1* and *swi7* mutants are sensitive to an HDAC inhibitor, Trichostatin. A(TSA)Ten-fold serial dilutions of wild-type (JY746), *mcl1-101* (NYSPC41), and *swi7-H4* (TN310) were plated onto YES containing 0 or 30 µg/ml of TSA and incubated for 4 days at the permissive (25 °C) or semi-permissive (33 °C) temperature.
(0.51 MB TIF)

Figure S4 Comparison of acetylation level between wild-type strain and *mcl1* mutant. ChIP-on-chip was performed as described in Materials and Methods. Values obtained from AcH4-KN were normalized to those obtained from the antibody that recognizes amino acid 25-28 of histone H4 in wild-type strain (A) or *mcl1-101* mutant (B), respectively. Similar results were observed in all chromosomes and the representative results in subtelomeric and centromeric regions of chromosome I were shown.
(1.63 MB TIF)

Table S1 (0.12 MB DOC)

Table S2 (0.05 MB DOC)
