# Supplementary figures and images for: A DNA Polymerase α Accessory Protein, Mcl1, Is Required for Propagation of Centromere Structures in Fission Yeast
Source: PLoS One. 2008 May 21;3(5):e2221. doi: 10.1371/journal.pone.0002221 (PMC2376062; doi:10.1371/journal.pone.0002221)

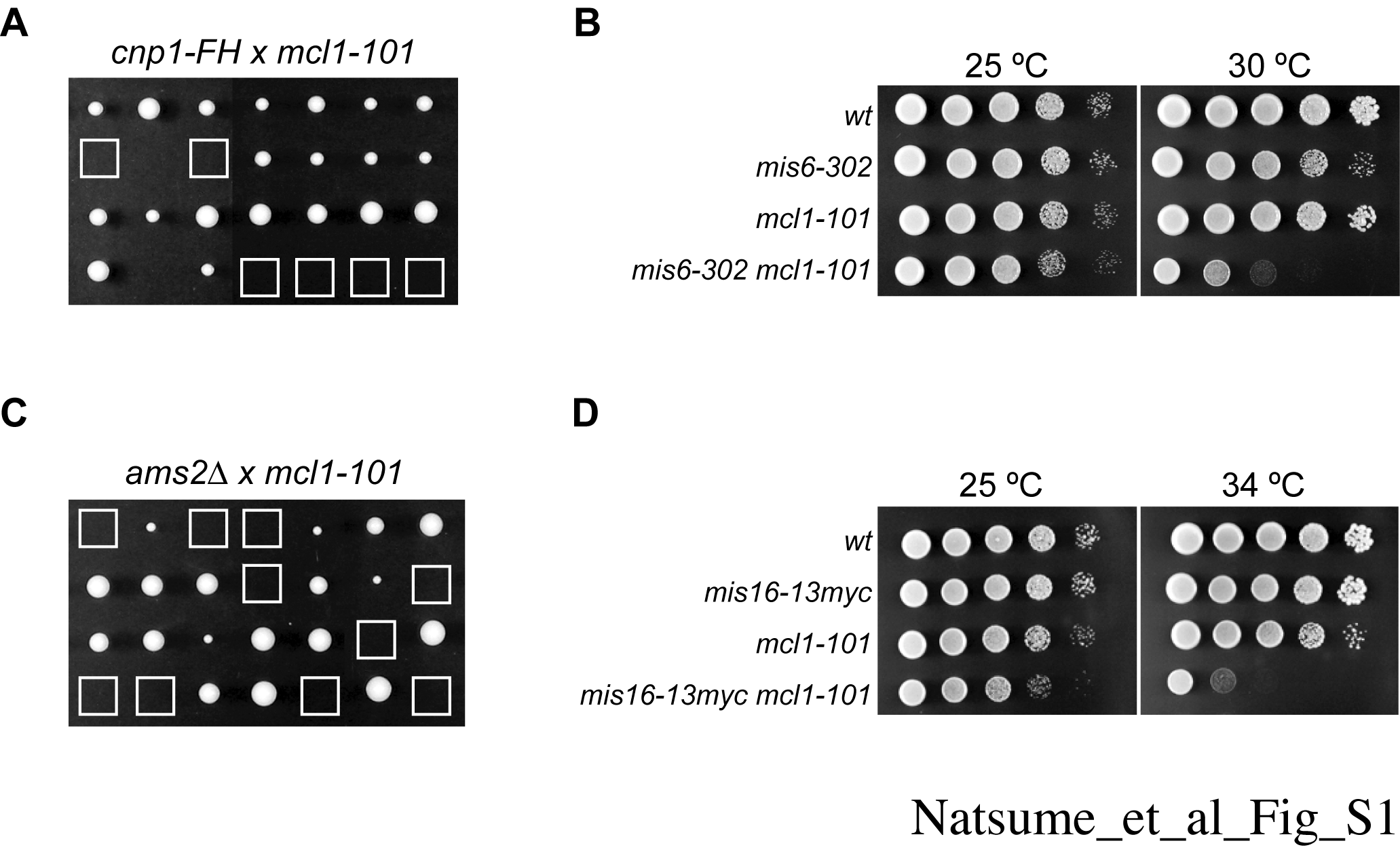

Supplement: Figure S1 — The mcl1-101 mutant interacts with kinetochore mutants genetically. (A) The mcl1-101 mutation is synthetically lethal with cnp1-FH. The mcl1-101 mutant (NYSPC40) was crossed with cnp1-FH (TN705). Resultant asci were dissected and incubated at 25 °C. Open squares indicate locations of double mutant spores. (B) Permissive growth temperature of mcl1-101 mutant is decreased by the mis6-302 mutation. Cells were grown at 25 °C and ten-fold serial dilutions were plated onto YES plates. Plates were incubated at indicated temperature for 3 days. Strains were derived from a cross between mcl1-101 (NYSPC41) and mis6-302 (NYSPL58). (C) The mcl1-101 mutation is synthetically lethal with ams2Δ mutation. The mcl1-101 mutant (NYSPC40) was crossed with ams2Δ (NYSPK66) as described in A. (D) Permissive growth temperature of mcl1-101 mutant is decreased by the mis16-13myc allele. Strains were wild-type (TN212), mis16-13myc (TN968), mcl1-101 (NYSPC41), mis16-13myc mcl1-101 (TN1035). (0.70 MB TIF) [file pone.0002221.s001.tif]

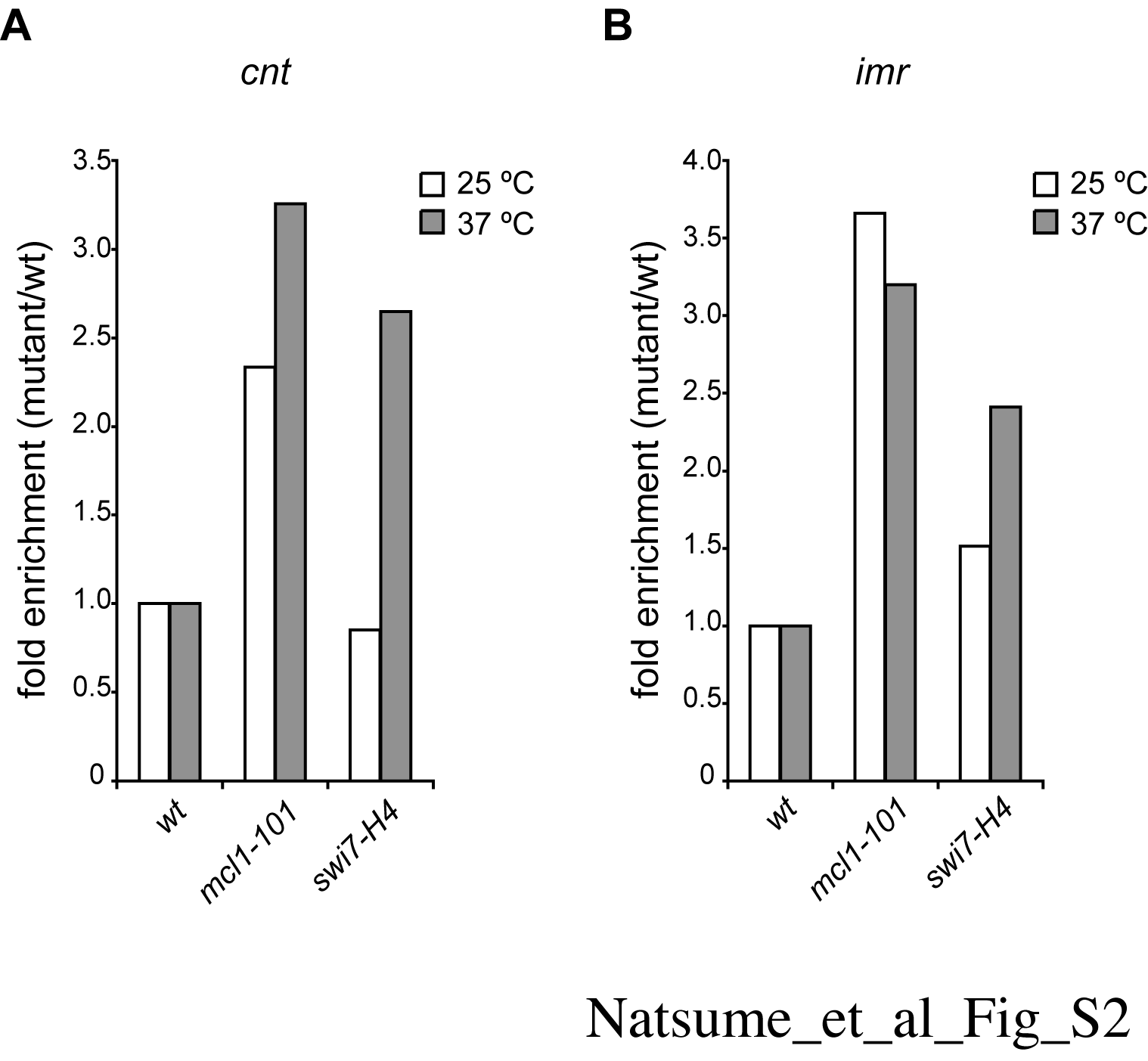

Supplement: Figure S2 — Histone H3 is aberrantly incorporated into kinetochore domain. ChIP was performed using antibody against C-terminal part of human histone H3. The ratio of immunoprecipitated DNA to input DNA was calculated and normalized to that of euchromatic lys1 locus. Fold enrichment compared to wild-type is shown. Open bars and shaded bars indicate results of 25 °C and 37 °C, respectively. Strains were wild-type (JY879), mcl1-101 (NYSPC52), swi7-H4 (TN403). (0.10 MB TIF) [file pone.0002221.s002.tif]

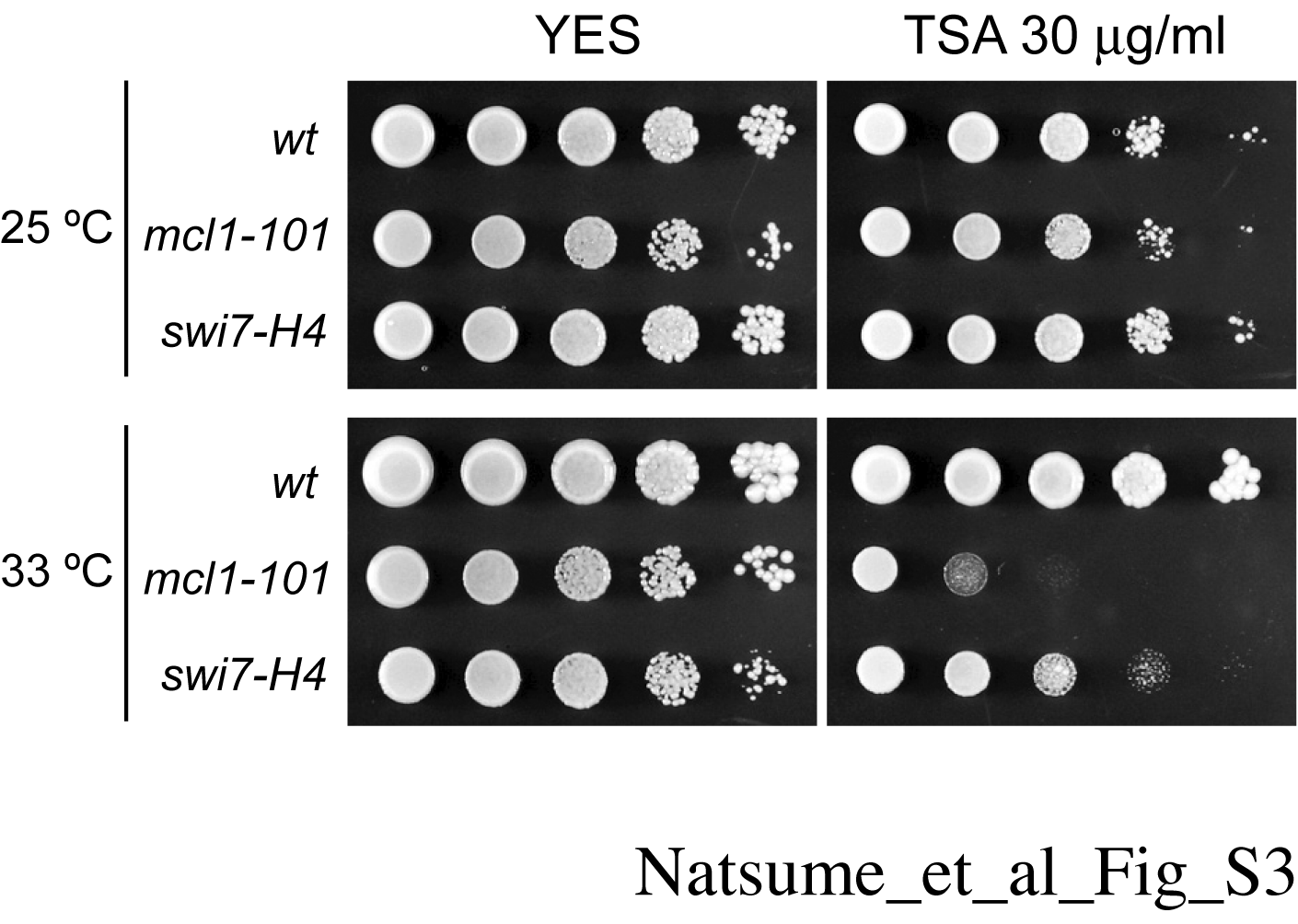

Supplement: Figure S3 — The mcl1 and swi7 mutants are sensitive to an HDAC inhibitor, Trichostatin. A(TSA)Ten-fold serial dilutions of wild-type (JY746), mcl1-101 (NYSPC41), and swi7-H4 (TN310) were plated onto YES containing 0 or 30 µg/ml of TSA and incubated for 4 days at the permissive (25 °C) or semi-permissive (33 °C) temperature. (0.51 MB TIF) [file pone.0002221.s003.tif]

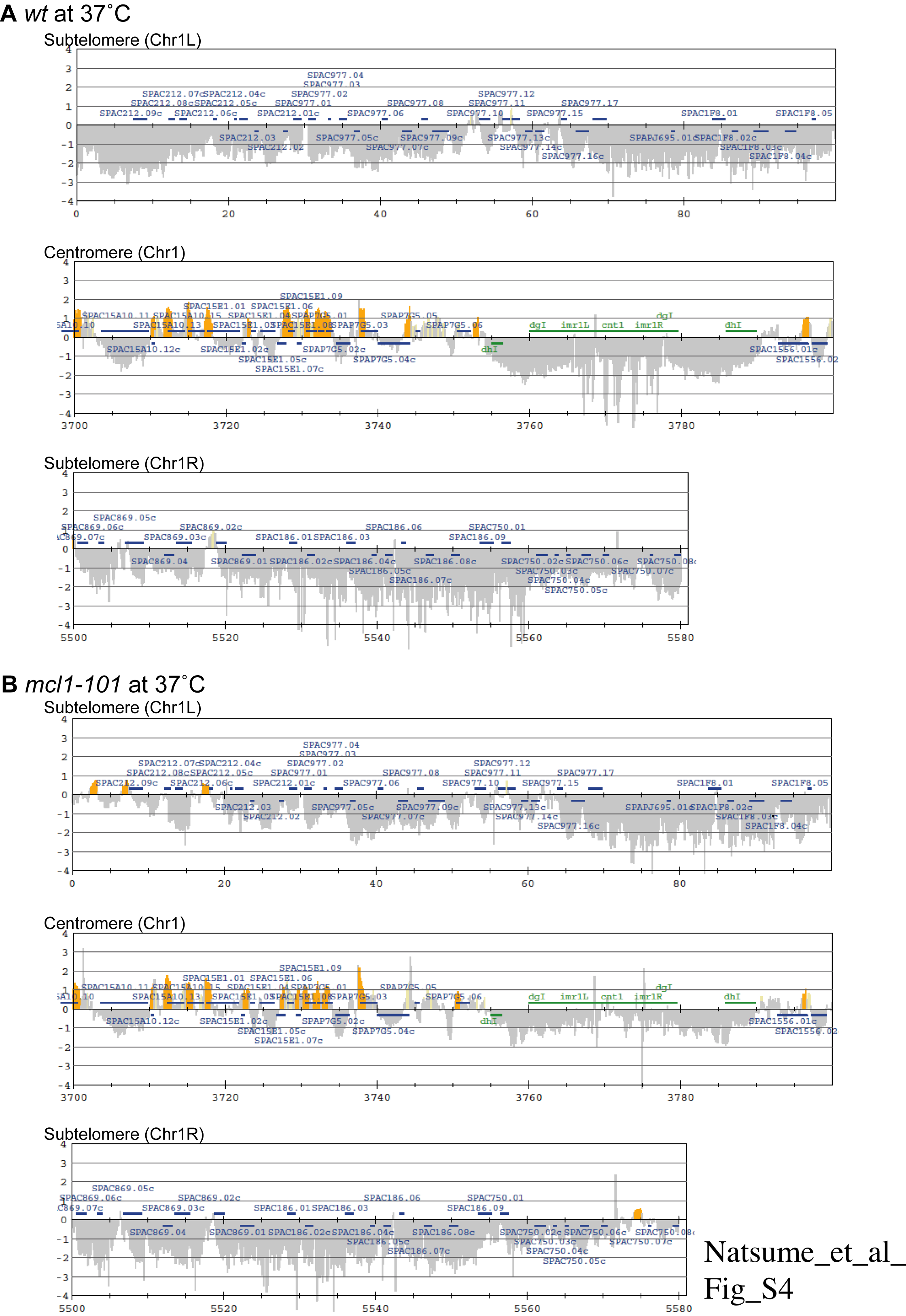

Supplement: Figure S4 — Comparison of acetylation level between wild-type strain and mcl1 mutant. ChIP-on-chip was performed as described in Materials and Methods. Values obtained from AcH4-KN were normalized to those obtained from the antibody that recognizes amino acid 25-28 of histone H4 in wild-type strain (A) or mcl1-101 mutant (B), respectively. Similar results were observed in all chromosomes and the representative results in subtelomeric and centromeric regions of chromosome I were shown. (1.63 MB TIF) [file pone.0002221.s004.tif]
