# Supplementary material for: A DNA Polymerase α Accessory Protein, Mcl1, Is Required for Propagation of Centromere Structures in Fission Yeast
Source: PLoS One. 2008 May 21;3(5):e2221. doi: 10.1371/journal.pone.0002221 (PMC2376062; doi:10.1371/journal.pone.0002221)
Supplement: Table S1 — (0.12 MB DOC) [file pone.0002221.s005.doc]

**Table S1**. Strains used in this study

| **Strain** | **Genotype** | **Source or reference** |
| --- | --- | --- |
| 972  JY746  JY741  JY879  Sp154  *swi7-H4*  *swi6Δ*  FY8452  FY336  FY3027  FY1180  FY1193  Hu78  *cos1-86*  *cos1-7*  *cos1-17*  *cos1-22*  *cos1-38*  *cos1-86*  *cos1-7*  *cos1-22*  *cos1-38*  TN212  NYSPC41  NYSPC40  NYSPC52  NYSPK66  TN310  TN403  NYSPL59  NYSPL58  TN667  TN829  NYSPC49  TN317  TN202  TN705  TN968  TN1035  NYSPI62  TN1217  TN1220  TN1226  TN1229  TN1173  TN1208  TN1214  TN1262 | *h*^-^  *h*^+^ *leu1-32 ura4-D18 ade6-M210*  *h*^-^ *leu1-32 ura4-D18 ade6-M216*  *h*^90^ *leu1-32 ura4-D18 ade6-M210*  *h*^-^ *leu1-32 ura4::ECFP-pcn1*^+^  *h*^-^ *leu1-32 swi7-H4*  *h*^+^ *swi6Δ::kan*^R^  *h*^-^ *leu1-32 ura4-D18 mis6-302*  *h*^-^ *leu1-32 ura4-DS/E ade6-M210 cnt1/TM1(NcoI)::ura4*^+^  *h*^+^ *leu1-32 ura4-D18 ade6-M210 arg3-D4 his3-D1 cnt1(NcoI)::arg3*^+^ *cnt3(NcoI)::ade6*^+^ *otr2(HindIII)::ura4*^+^ *tel1L::his3*^+^  *h*^+^ *leu1-32 ura4-D18 ade6-M210 otr1R(SphI)::ade6*^+^  *h*^+^ *leu1-32 ura4-D18 ade6-M210 imr1L(NcoI)::ura4*^+^ *otr1R(SphI)::ade6*^+^  *h*^+^ *leu1-32 ura4- ade6-M210 otr1R(SphI)::ade6*^+^ *rik1Δ::LEU2*  FY3027 *cos1-86*  FY3027 *cos1-7*  FY3027 *cos1-17*  FY3027 *cos1-22*  FY3027 *cos1-38*  FY1180 *cos1-86*  FY1180 *cos1-7*  FY1180 *cos1-22*  FY1180 *cos1-38*  *h*^-^ *leu1-32 ura4-D18 ade6-M210*  *h*^+^ *leu1-32 ura4-D18 ade6-M210 mcl1-101*  *h*^-^ *leu1-32 ura4-D18 ade6-M210 mcl1-101*  *h*^90^ *leu1-32 ura4-D18 ade6-M210 mcl1-101*  *h*^+^ *leu1-32 ura4-D18 ade6-M210 ams2Δ::kan*^R^  *h*^+^ *leu1-32 ura4-D18 ade6-M210 swi7-H4*  *h*^90^ *leu1-32 ura4-D18 ade6-M210 swi7-H4*  *h*^+^ *leu1-32 ura4-D18 ade6-M210 mis6-302*  *h*^-^ *leu1-32 ura4-D18 ade6-M210 mis6-302*  *h*^-^ *leu1-32 ura4-DS/E ade6-M210 cnt1/TM1(NcoI)::ura4*^+^ *mcl1-101*  *h*^+^ *leu1-32 ura4-D18 ade6-M210 cnt1/TM1(NcoI)::ura4*^+^ *swi7-H4*  *h*^+^ *leu1-32 ura4-D18 ade6-M210 imr1L(NcoI)::ura4*^+^ *otr1R(SphI)::ade6*^+^ *mcl1-101*  *h*^+^ *leu1-32 ura4-D18 ade6-M210 imr1L(NcoI)::ura4*^+^ *otr1R(SphI)::ade6*^+^ *swi7-H4*  *h*^-^ *leu1-32 ura4-D18 ade6-M210 imr1L(NcoI)::ura4*^+^ *otr1R(SphI)::ade6*^+^ *swi6Δ::kan*^R^  *h*^+^ *leu1-32 ura4-D18 ade6-M210 cnp1-FH::kanMX6*  *h*^-^ *leu1-32 ura4-D18 ade6-M210 mis16-13myc::kanMX6*  *h*^-^ *leu1-32 ura4-D18 ade6-M210 mis16-13myc::kanMX6 mcl1-101*  *h*^+^ *leu1-32 ura4-D18 ade6-M210 mcl1-GFP::kanMX6*  *h*^-^ *mcl1-Venus::kanMX6*  *h*^-^ *mcl1-ECFP::kanMX6*  *h*^-^ *swi7-Venus::kanMX6*  *h*^-^ *swi7-CFP::kanMX6*  *h*^-^ *swi7-GFP::kanMX6*  *h*^-^ *mcl1-GFP::kanMX6 ura4::ECFP-pcn1*^+^  *h*^-^ *swi7-GFP::kanMX6 ura4::ECFP-pcn1*^+^  *h*^-^ *mcl1-ECFP::kanMX6 swi7-Venus::kanMX6* | Lab stock  M. Yamamoto  M. Yamamoto  M. Yamamoto  G. Baldacci  H. Okayama  O. Niwa  YGRC^a^  [26]  [22]  [26]  [19]  [26]  This study  This study  This study  This study  This study  This study  This study  This study  This study  This study  [29]  This study  This study  This study  This study  This study  This study  This study  This study  This study  This study  This study  This study  This study  This study  This study  This study  This study  This study  This study  This study  This study  This study  This study  This study |

^a^ Yeast Genetic Resource Center (YGRC) in National Bio-Resource Project, Japan
