# Supplementary material for: A DNA Polymerase α Accessory Protein, Mcl1, Is Required for Propagation of Centromere Structures in Fission Yeast
Source: PLoS One. 2008 May 21;3(5):e2221. doi: 10.1371/journal.pone.0002221 (PMC2376062; doi:10.1371/journal.pone.0002221)
Supplement: Table S2 — (0.05 MB DOC) [file pone.0002221.s006.doc]

**Table S2**. Primers used in this study

| **Primer Name** | **Sequence** |  |
| --- | --- | --- |
| Venus-F0  Venus-R0  Ams2-F0  Ams2-R0  Sir2-F0  Sir2-R0  Clr3-F0  Clr3-R0  KanMX6-F0  KanMX6-R0  cnt1-F1  cnt-R1  imr1L-F1  imr1L-R1  otr1L-F1  otr1L-R1  cnt1-1  cnt1-2  imr-1  imr-2  lys1-1  lys1-2 | AGTCTTAATTAACACCATGGTGAGCAAGGG  GACTGGCGCGCCTTACTTGTACAGCTCGTC  CTTGGAAAAGTTCAGCATCC  ATAAGGATGTGGCTTTGTCC  AGTCCTGCAGATGTATTTCAAATGAGAAAGTTC  GACTCTGCAGTTTGGTGGCTTTTTCGATGATC  AGCTGGATCCCATCCTTCATTTTAGATGGTATAC  GATCGGATCCAAATCAATTTTTTTGTGTGATATAG  CGGATCCCCGGGTTAATTAA  ATGCCCCGGGGAATTCGAGCTCGTTTAAAC  ATGCGAATTCAACTCTGTTTATTAA  TAGAAAGCTTTAATTATTCGTTCG  ATGTGCATGCTATATTGGTATTG  TACCAAGCTTTACTAAATGTAAGAA  CAGGGCATGCTTAGCAAGTAC  ATTTAAGCTTTCCCTTGAATTTTC  CACAACCATAAAGCCGTATTC  AACCCCGTTGTTAAACATATG  AGCATTGCCAGCTCATATTA  GAGCTTATGGAAAAAATGTTTG  CCAACAGATTATAGTCGTCC  CTTGAGCAGCAACATAAACC | Cloning  MNase assay  qPCR |
